# Giant Breast Lipoma: A Case Report

**DOI:** 10.7759/cureus.22304

**Published:** 2022-02-16

**Authors:** Zaki Busbaih, Ali A Almohammed Saleh, Maitha K AlMaghlouth, Abdulqader M Albeladi, Tayseer Alali, Mohammad S AlGhadeer, Ahmad Odeh

**Affiliations:** 1 General Surgery, Prince Saud Bin Jalawi Hospital, Al-Mubarraz, SAU; 2 Medical School, King Faisal University, Hofuf, SAU; 3 General Surgery and Laparoscopy, Prince Saud Bin Jalawi Hospital, Al-Mubarraz, SAU

**Keywords:** excision surgery, lipoma, breast mass, giant lipoma, benign breast condition

## Abstract

Lipomas are slow-growing, benign mesenchymal masses. Most lipomas are small, weighing only a few grams; however, if their size becomes exceptionally large, they are called giant lipomas. Giant lipoma of the breast is infrequently observed due to the rarity of size and location, with very few case reports available in the literature. Here, we report the case of a 48-year-old female patient who presented with a painless, huge lump in her right breast. The patient underwent surgical removal of the mass with a histologic examination confirming the diagnosis of a giant breast lipoma.

## Introduction

Lipoma is one of the most common benign neoplasms, derived from fatty tissue, and represents 16% of all mesenchymal tumors [1]. Lipomas usually occur in the age group of 40-60 years. They are slow-growing, without pain or functional impairment [2]. Lipomas are usually freely mobile underneath the skin and develops as well-circumscribed, encapsulated masses [3]. They are mostly small (less than 5 cm in diameter) with a weight of a few grams only [4]. They could occur in any part of the body and 20% are located in the chest wall [5]. Breast lipomas are usually small and may be difficult to diagnose due to the normal adipose tissue of the breast [6]. When the diameter of a lipoma is at least 10 cm or its weight exceeds 1,000 grams, it is considered as giant lipoma [7]. A giant breast lipoma is a rare manifestation so making a correct diagnosis is crucial in the prevention of the overtreatment of this mass, which otherwise may be managed as a malignant tumor. Here, we report a rare case of a 48-year-old female patient with a giant lipoma in the right breast measuring 30 x 18 x 3 cm causing breast asymmetry.

## Case presentation

A 48-year-old female presented to the surgery clinic with a painless lump in her right breast for three years. The lump grew gradually in size. The patient is medically free with no previous history of breast trauma or disease. She has a history of tongue papilloma that was excised five years ago. She did not have a family history of breast carcinoma. Physical examination of the right breast revealed a well-defined, homogeneous, firm, non-tender, mobile mass, measuring approximately 30 × 18 × 3 cm in diameter, with dilated veins on the surface. The overlying skin of the mass and the nipple-areola complex appeared to be normal. There was no nipple discharge. Axillary lymphadenopathy was not present. The left breast examination was unremarkable.

**Figure 1:**
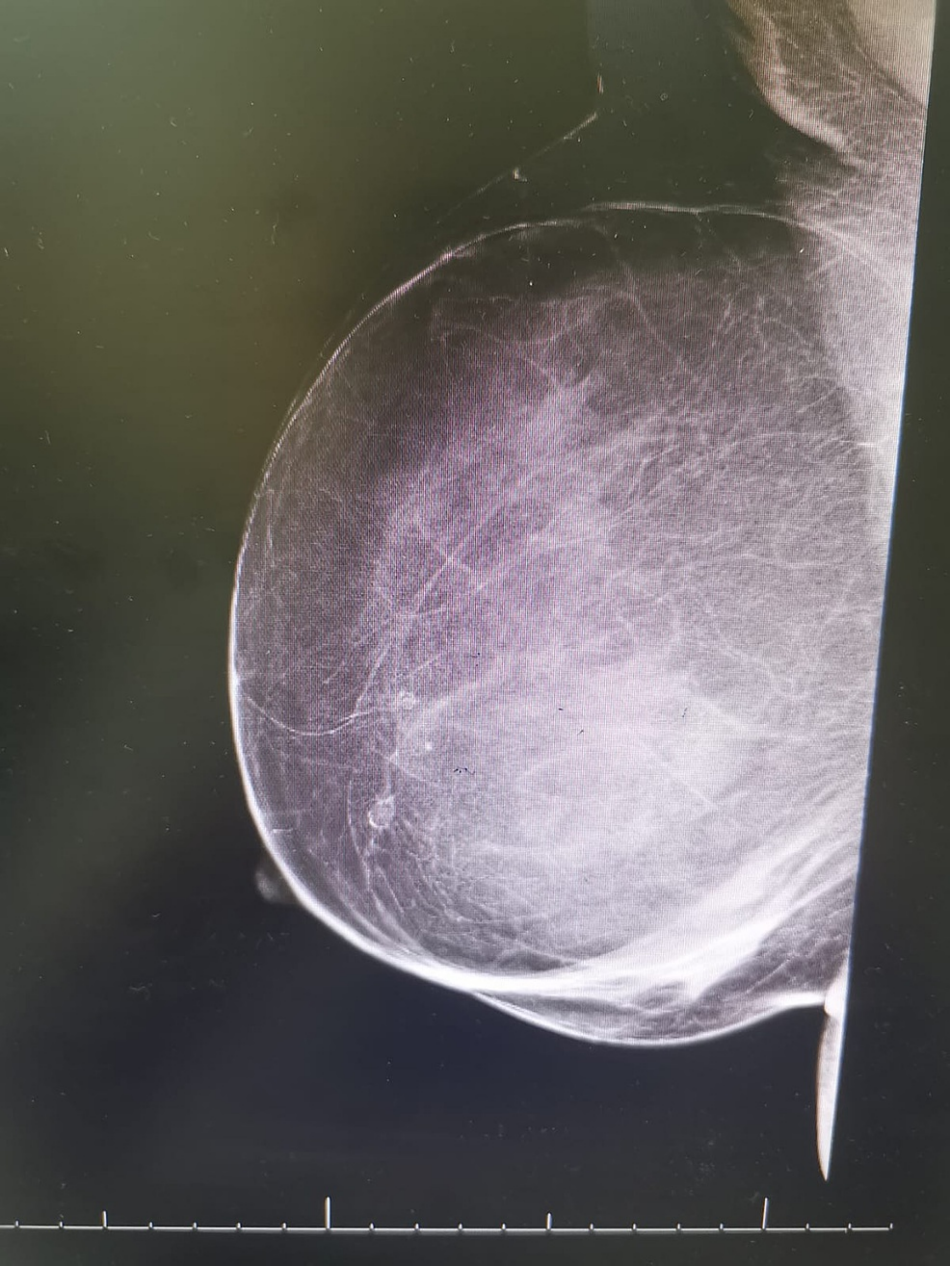
Mammographic view of the right breast shows several round, well-circumscribed, encapsulated, radiolucent masses.

Mammogram images revealed a right breast replacement by a huge homogeneously isoechoic encapsulated mass with an internal tiny cyst with peripheral calcification measuring about 16 × 14 cm causing medial compression of the breast tissue. No skin thickening or nipple retraction was observed. There was no intralesional vascularity or enlarged axillary lymph nodes detected (Figure 1).

In correlation to the mammogram, it represented a large lipoma with central fat necrosis, consistent with breast imaging-reporting and data system (BI-RADS) assessment category 2, showing benign findings. Fine needle aspiration cytology (FNAC) was not performed as it was not needed.

**Figure 2:**
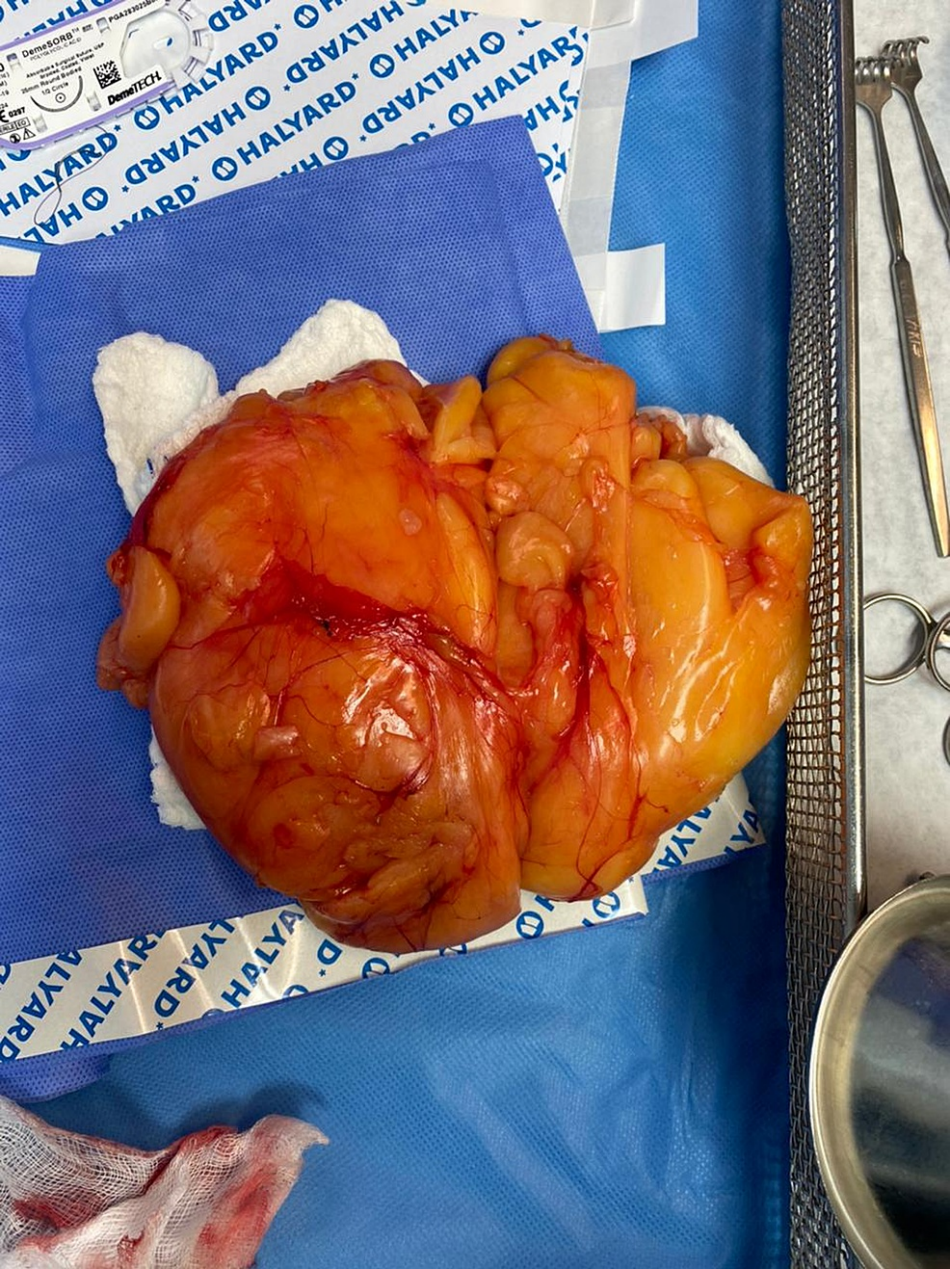
Gross appearance of the mass.

An elective surgical excision was scheduled and performed under general anesthesia. The patient was placed in the supine position on the table; aseptic technique was followed, and draping was provided. The right breast revealed an intracapsular, lobular, solitary mass. A skin incision was made around the areola followed by dissection around the lipoma to excise it. The mass weighed 1000 g (Figure 2).

Hemostasis was achieved with electrocautery. Washing and drain insertion were applied under negative pressure. Closure was done intradermally with a subcuticular suture, and a pressure dressing was applied. Histopathologic examination of the specimen revealed one huge soft consistency, irregular surface, and yellow-colored tissue measuring 30 × 18 × 3 cm. Cut sections showed adipose tissue without evidence of atypia or malignant change. The findings were consistent with lipoma of the breast. The patient had an uneventful postoperative course. She was discharged the next day after drain removal, to be followed up in the outpatient clinic.

## Discussion

Lipomas are one of the most common adipose tumors. They are usually benign, well-circumscribed, and covered by a thin capsule [8]. The etiology of lipomas remains unidentified. However, it has been shown to be sporadic or linked to an inherited condition [9,10]. They can arise in any part of the body, with a prevalence rate of 2.1 per 1000 people [9]. Of lipomas, 20% are found in the chest wall, and the breast is a common site for this pathology [5,11]. They vary in size with the vast majority being small in size weighing only a few grams [11]. Hawary et al. defined a giant breast lipoma by the presence of a lesion with a dimension of at least 5 cm and a weight more than 500 g [12]. However, according to Sanchez et al., a giant lipoma has a dimension of 10 cm [7]. A mass that weighed 5700 g and measured 35 x23 x20 cm, reported by Ribeiro et al., appears to be the largest in literature [13]. Lipomas are usually solitary but might be hard to detect in large or postmenopausal breasts [14].

Patients typically present when lipomas cause anatomical changes that may lead to cosmetic concerns or interfere with function [15]. The presence of size disparity and architectural distortion mandates a comprehensive history, clinical examination, and investigations [16]. Because of the post-traumatic fat necrosis, lipomas may also appear as firm, fixed masses that may be clinically misinterpreted as malignancies [17]. Imaging techniques such as ultrasonography and mammography prove to be beneficial and useful for a more accurate diagnosis, especially in tailoring a specific management plan for every patient [6]. On mammography, a radiolucent nodule surrounded by a thin radiopaque capsule that may include ring-like calcifications owing to fat necrosis can establish the diagnosis of a lipoma. A correct diagnosis is critical since breast lipomas can be misdiagnosed as malignancies, phyllodes tumors, fibroadenomas, and duct papillomas [18]. Achieving the correct diagnosis might be challenging, necessitating an invasive approach such as FNAC or punch biopsy [17]. According to Lanng et al, the diagnosis of breast lipoma can be a diagnostic dilemma with difficulties in establishing the diagnosis through clinical examination, imaging studies, and FNAC [1]. In our case, FNAC was not done as the diagnosis was established according to the mammogram findings and confirmed through the histopathological examination that was done after the excision of the mass. Santi et al. reported a similar case of a 27-year-old female who presented with significant breast asymmetry. Preoperatively, she was diagnosed with breast liposarcoma. After excision and histopathology study was done, the result revealed the presence of a giant breast lipoma measuring 15.5 ×9.3 ×4.5 cm and weighing 257 grams; on histopathology, a uniform fatty composition with benign adipocytes was discovered [19].

Surgical excision has been the mainstay of treatment for lipoma historically. However, advanced medical treatment might cause a reduction in lipoma size. Two to three injections of deoxycholate have shown a decrease in approximately 75% of the lesion size in a case series [20]. Liposuction-assisted excision could be also considered as another definitive management of lipoma [7]. This technique is preferred in areas where larger scars should be avoided because it allows the incision to be placed in an inconspicuous location [21]. Although it's associated with better cosmetic results, it has been shown to increase the risk of recurrence and hematoma formation due to incomplete capsule removal [7]. Furthermore, skin irregularities such as dimpling, numbness, paresthesia, and pigmentation changes are all common complications after the liposuction technique [22-23]. The main surgical indications for breast lipoma removal include cosmetic deformity, patient discomfort, and the rapid growth of the lump [15]. For many uncomplicated cases, the excision can be performed in the minor operating room under local anesthesia [24]. However, since our patient presented with a huge mass that may have invaded the deep fascia or muscles, we decided to manage the patient with an advanced procedure under general anesthesia. The skin incisions should follow the langer's lines and be elliptical in order to obtain the best cosmetic result [24]. Hematomas and seromas are considered the most common postoperative complications [25]. Giant breast lipomas always cause discomfort and heaviness in patients. So, surgical intervention is often required for cosmetic reasons or to alleviate the patient's symptoms [15].

## Conclusions

Breast lipomas are usually observed when they reach considerable dimensions and produce breast asymmetries and cosmetic problems that determine the patient's need to proceed with surgery. Whenever breast lipomas are diagnosed, they should be entirely excised and sent for histopathological studies in order to confirm a positive diagnosis of benignity. Long-term follow-up is necessary since lipomas can relapse even after several years.
